# Novel Approaches to the Management of Myelodysplastic Syndromes: The Roles of Artificial Intelligence and Oxidative Stress Biomarkers

**DOI:** 10.3390/hematolrep18030033

**Published:** 2026-05-15

**Authors:** Ioannis Tsamesidis, Georgios Drillis, Sotirios Varlamis, Niki Smaragdaki, Philippos Klonizakis, Maria Dimou, Konstantinos Liapis, Georgios Vrahiolias, Eleni Andreadou, Stella Mitka, Maria Chatzidimitriou, Ioannis Kotsianidis, Petros Skepastianos, Anastasios G. Kriebardis, Ilias Pessach

**Affiliations:** 1 Department of Biomedical Sciences, Faculty of Health Sciences, International Hellenic University, Sindos, 57400 Thessaloniki, Greece; svarlamis@ihu.gr (S.V.); nikismaragdaki@gmail.com (N.S.); eandreadou@ihu.gr (E.A.); chdimitr@ihu.gr (M.C.); pskep@otenet.gr (P.S.); 2 Laboratory of Reliability and Quality Control in Laboratory Hematology (HemQcR), Department of Biomedical Sciences, School of Health & Caring Sciences, University of West Attica (UniWA), 12243 Egaleo, Greece; gdryllis@uniwa.gr (G.D.);; 3 Clinical Research and Evidence-Based Medicine Unit, Second Medical Department, Aristotle University of Thessaloniki, 54642 Thessaloniki, Greece; philklon@auth.gr (P.K.); koliapi@med.duth.gr (K.L.); george_vrachiolias@yahoo.com (G.V.); 4 Department of Hematology and Bone Marrow Transplantation, ‘Laikon’ General Hospital, National and Kapodistrian University of Athens, 15772 Athens, Greece; msdimou@med.uoa.gr; 5 Department of Hematology, University Hospital of Alexandroupolis, Democritus University of Thrace, 68100 Alexandroupolis, Greece; kotsianidis@hematology-pgna.gr

**Keywords:** artificial intelligence, machine learning, deep learning, myelodysplastic syndromes, oxidative stress biomarkers

## Abstract

**Objectives**: Myelodysplastic syndromes (MDSs) are a heterogeneous group of clonal hematopoietic disorders characterized by ineffective hematopoiesis, genomic instability, and a high risk of progression to acute myeloid leukemia. Oxidative stress (OS) has emerged as a central factor in MDS pathophysiology, contributing to DNA damage, altered cellular signaling, and disease progression. Recent advances in artificial intelligence (AI) and machine learning (ML) offer a transformative approach for integrating multidimensional datasets including oxidative stress markers, hematologic parameters, and molecular profiles to enhance diagnosis, prognostication, and therapeutic monitoring in MDS. **Methods**: A comprehensive literature search was conducted in PubMed and Scopus, using the keywords “OS biomarkers,” “AI,” and “MDS’’. **Results**: Modified redox biomarkers can be correlated with oxidative imbalance and disease progression. ML models such as neural networks, decision trees, and support vector machines effectively capture complex relationships among redox biomarkers, enhancing risk stratification and prediction of treatment response. AI-driven proteomic analyses further revealed OS-related protein signatures linked to MDS pathophysiology. Overall, AI and ML enable the transformation of multidimensional OS data into clinically actionable tools for personalized management in MDS. **Conclusions**: Integrating biomarker research with AI-based analytics holds promise for advancing personalized diagnostics, prognostication, and therapeutic strategies in MDS, paving the way toward precision medicine.

## 1. Introduction

Myelodysplastic syndrome (MDS) is frequently characterized by inefficient hematopoiesis, dysplasia in one or more myeloid cell lineages, variable degrees and number of cytopenias, and increased risk of progression to acute myeloid leukemia (AML) [1]. MDS is a pre-malignant hematopoietic disorder that transforms to AML in approximately 35% of cases [2]. It is also well known that 40% of MDS patients show cytogenetical alterations at diagnosis [3,4]. Oxidative stress (OS) caused by excessive reactive oxygen species (ROS) is likely to affect the functions of hematopoietic stem cells, such as cell growth and self-renewal, which may contribute to defective hematopoiesis [5,6]. In detail, OS results from an imbalance between reactive oxygen species (ROS) production and antioxidant defenses, leading to cellular damage and has been implicated in various diseases. Several biomarkers have been identified to assess oxidative stress levels [7,8,9,10]. One such marker is 8-oxo-2′-deoxyguanosine (8-oxo-dG), a product of DNA oxidation, which accumulates in tissues under oxidative stress conditions [11]. Elevated 8-oxo-dG levels have been associated with increased cancer risk and other pathologies [12]. Another important biomarker is 9-hydroxyoctadecadienoic acid (9-HODE), derived from the oxidation of linoleic acid [13]. Increased 9-HODE levels have been observed in diseases such as cataracts, rheumatoid arthritis [14], and Alzheimer’s disease, indicating its potential as a marker for oxidative-stress-related conditions [15]. Additionally, protein carbonylation, resulting from protein oxidation, serves as a reliable indicator of oxidative stress [16]. Elevated protein carbonyl levels have been linked to aging and various diseases, including neurodegenerative disorders and diabetes [17,18]. These biomarkers are instrumental in evaluating oxidative stress and understanding its role in disease development [7,19]. ROS regulate essential cellular processes such as proliferation, differentiation, and apoptosis; however, excessive ROS production disrupts redox homeostasis, contributing to disease progression and treatment resistance and has been implicated in the development of several types of cancer, including MDS [1]. At the molecular level, redox homeostasis is tightly regulated by signaling pathways such as the KEAP1 (Kelch-like ECH-associated protein 1)–NRF2 (Nuclear factor erythroid 2-related factor 2) axis. Under basal conditions, NRF2 is sequestered in the cytoplasm by KEAP1 and targeted for degradation. In response to oxidative stress, NRF2 is released and translocates to the nucleus, where it activates the transcription of antioxidant and cytoprotective genes. Dysregulation of this pathway may contribute to impaired oxidative stress responses in MDS, potentially influencing disease progression and therapeutic resistance. Incorporating such pathway-level insights may enhance the biological interpretation of oxidative stress biomarkers, particularly when integrated with AI-based analytical approaches. A study by Tsamesidis et al. [20] evaluated oxidative stress biomarkers in patients with different hematological malignancies, including MDS. The study found that patients with MDS exhibited elevated levels of reactive oxygen species (ROS) and malondialdehyde (MDA), along with decreased total antioxidant capacity (TAC) and vitamin E levels, compared to healthy controls. Indeed, an increase in proinflammatory cytokines was already established in the MDS bone marrow microenvironment, leading to an ultimate ROS increase in MDS cells. Oxidative stress affects the self-renewal, proliferation and differentiation of hematopoietic cells (https://pmc.ncbi.nlm.nih.gov/articles/PMC9836226/, accessed on 5 November 2025). These findings suggest that oxidative stress plays a significant role in MDS and that these biomarkers could serve as potential indicators for disease progression. The Revised International Prognostic Scoring System (IPSS-R) is the most frequently used prognostic system and is based on a small number of features with independent prognostic value, including chromosomal abnormalities, bone marrow blasts, hemoglobin level, platelet count and absolute neutrophil count. These features allow the classification of MDS patients into five risk subgroups (very low risk, low risk, intermediate risk, high risk, and very high risk) with different probabilities of AML progression and survival. Lower-risk MDS (IPSS-R risk score ≤ 3.5) accounts for about two-thirds of all MDS cases [1]. Recent advances in artificial intelligence (AI) have contributed to a deeper understanding of the disease and its progression, enabling the development of refined prognostic models. Techniques such as convolutional neural networks (CNNs), XGBoost, and random forest have been employed to analyze large datasets, integrating clinical, molecular, and pathological information. Through these advanced models, researchers are able to improve prognostic accuracy for MDS patients.

The objective of this review is to examine the current evidence on the role of oxidative stress biomarkers in myelodysplastic syndromes (MDSs) and to evaluate how artificial intelligence (AI) and machine learning (ML) approaches can integrate these biomarkers with clinical and molecular data to improve diagnosis, prognostic assessment, and disease monitoring. Furthermore, this review discusses current challenges, methodological limitations, and future perspectives for the implementation of AI-driven biomarker analysis in the management of MDS.

## 2. Materials and Methods

A comprehensive literature search was conducted using multiple electronic databases, including PubMed/MEDLINE, Scopus, Web of Science, and Google Scholar. Additional sources such as the Cochrane Library and EMBASE were also screened to ensure broad coverage of relevant studies. The search strategy combined keywords and Boolean operators, including: “myelodysplastic syndromes” OR “MDS,” AND “oxidative stress” OR “reactive oxygen species” OR “malondialdehyde” OR “8-OHdG” OR “antioxidant enzymes,” AND “artificial intelligence” OR “machine learning” OR “deep learning.” Additional combinations such as “AI-driven diagnostics AND MDS” and “machine learning AND hematological malignancies” were also applied. Studies were included if they (i) investigated oxidative stress biomarkers in MDS or related hematological disorders, (ii) evaluated artificial intelligence or machine learning approaches in diagnosis, prognosis, or disease modeling, or (iii) provided mechanistic insights into redox regulation relevant to MDS. Both circulating (serum/plasma) and intracellular (cellular, including bone marrow or peripheral blood cells) biomarkers were considered. Exclusion criteria included: (i) non-English publications, (ii) conference abstracts without full text, (iii) studies lacking sufficient methodological detail, and (iv) studies not directly relevant to oxidative stress or AI applications in hematological conditions.

The initial search yielded 62 records. After the removal of duplicates and screening of titles and abstracts, 45 articles were selected for full-text review. Ultimately, 35 studies were included in this review based on relevance and quality.

## 3. Results and Discussion

Artificial intelligence (AI) is increasingly applied to the analysis of oxidative stress biomarkers, enabling rapid and precise evaluation of molecular alterations of redox signatures. Machine learning and deep learning approaches, including neural networks, decision trees, and regression models, can capture complex, non-linear interactions among oxidative stress parameters and improve prediction and classification tasks.

Although direct applications in myelodysplastic syndromes (MDSs) remain limited, studies in other clinical contexts illustrate the potential of these approaches. For example, AI-based composite indices integrating inflammatory and biochemical markers have been used to quantify systemic stress responses, suggesting that similar integrative models could be developed for risk stratification in MDS. Likewise, neural network models applied to antioxidant activity data in complex diseases demonstrate the ability of AI to resolve non-linear oxidative stress interactions, which may be relevant to the heterogeneous biology of MDS.

Additionally, the AI-driven integration of oxidative-stress-related biomarkers with clinical and molecular parameters has been shown to support personalized risk prediction in other fields, raising the possibility that such approaches could enhance prognostic modeling and therapeutic decision-making in MDS. However, the application of these methods in MDS requires disease-specific datasets, standardized biomarker selection, and rigorous clinical validation.

## 4. Part A: Role of AI and ML in Diagnosis of MDS

Although the studies discussed above highlight the broad applicability of artificial intelligence in analyzing oxidative stress biomarkers across different medical fields, the relevance of these approaches extends directly to hematological disorders such as myelodysplastic syndromes (MDSs). Oxidative stress has been recognized as a key contributor to MDS pathophysiology, influencing genomic instability, ineffective hematopoiesis, and disease progression. Therefore, integrating AI-based analytical methods with oxidative stress biomarker assessments may provide valuable insights into MDS diagnosis, prognostic stratification, and disease monitoring. The following section discusses current applications of AI and machine learning in the diagnosis and risk assessment of MDS. AI and ML can be used for the diagnosis of MDS and myeloid neoplasms by improving diagnostic accuracy, efficiency, and prognostic assessment. AI-driven techniques, such as deep-learning-based analysis of bone marrow morphology, peripheral blood samples and flow cytometry data, enable more precise detection of dysplastic and abnormal changes and can identify genetic mutations and biomarkers connected with myeloid neoplasms. Various machine learning models, including convolutional neural networks (CNNs), decision trees, random forests, Gradient Boosting Machines (GBMs), and ElasticNet, have been applied to analyze these data sources. The performance of these models has been evaluated using metrics such as the Area Under the Curve (AUC), accuracy, sensitivity, and specificity and it demonstrates their potential to enhance diagnostic reliability [21]. In detail, deep learning approaches, particularly convolutional neural networks (CNNs), are primarily used for image-based data such as bone marrow smears and peripheral blood morphology, where they excel at detecting subtle dysplastic features. In contrast, machine learning models such as random forests, Gradient Boosting Machines, and ElasticNet are more suitable for structured clinical, cytogenetic, and flow cytometry datasets, as they can handle heterogeneous variables and identify key predictive features. While ensemble methods (e.g., random forests and XGBoost) often demonstrate high predictive performance and robustness, they may lack interpretability, whereas simpler models such as decision trees offer greater transparency but lower accuracy. This highlights a trade-off between performance and clinical interpretability, which remains a key consideration for implementation in MDS.

Diagnosing MDS is challenging and depends on the combined assessment of cytological, cytogenetic, and molecular characteristics. The 2016 World Health Organization (WHO) classification considers cytomorphology and cytogenetics as the primary methods for diagnosing MDS. However, using bone marrow smears can be challenging, which is why additional techniques like next-generation sequencing (NGS) and multiparametric flow cytometry (MFC) are used to support diagnosis. However, these methods are not yet widely implemented due to inconsistencies in standardization as different laboratories use varying flow cytometry machines and antibody brands. Notably, the development of feature selection algorithms is making it possible to identify the most critical parameters for a more precise and reliable diagnosis [22].

**Figure 1:**
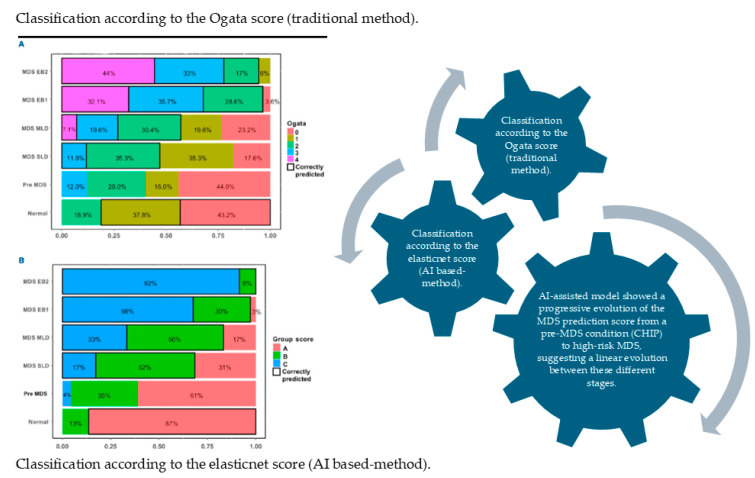
Distribution of MDS predictions across diagnostic groups using the Elastic Net MDS score and the Ogata score. The distribution of pathology subtypes is shown according to classification by the Ogata score and by the penalized logistic regression (Elastic Net) score. Diagnoses of myelodysplastic syndrome with single-lineage dysplasia (MDS-SLD) and multilineage dysplasia (MDS-MLD) were established by cytological assessment. (**A**) Classification based on the Ogata score. (**B**) Classification based on the Elastic Net score. MDS-EB1: MDS with excess blasts (5–9%); MDS-EB2: MDS with excess blasts (10–19%); pre-MDS: pre-MDS conditions. Adapted with permission from Ref [22]. Copyright 2023 Haematologica.

In a retrospective cohort of 60 Southeast Asian patients with MDS undergoing allogeneic stem cell transplantation, the study assessed various pre-transplant prognostic systems and clinical factors for their influence on transplant outcomes. Multivariate analysis revealed that the WHO classification-based Prognostic Scoring System (WPSS) was significantly associated with overall survival (OvS), progression-free survival (PFS), cumulative incidence of relapse (CIR), and non-relapse mortality (NRM). Stratified by WPSS risk category, 3-year OS rates were 100% (very low/low), 61%, 37%, and 18% (very high), while PFS, CIR, and NRM similarly showed graded differences across risk groups (e.g., higher relapse and non-relapse mortality in higher WPSS strata). Other predictors such as WHO subtype, IPSS-R cytogenetic risk, donor sex, and graft-versus-host disease also impacted specific outcomes. The authors concluded that the WPSS is a robust predictor of post-transplant outcomes in this population and suggested its use to guide risk-adapted strategies (e.g., intensified monitoring and adjunctive therapies) for higher-risk MDS patients [23]. While the WPSS has been shown to robustly predict post-transplant outcomes in Southeast Asian MDS patients, techniques integrating AI—such as machine-learning-based risk modeling—could further refine prognostication by combining WPSS scores with molecular, cytogenetic, and clinical data to generate more personalized, dynamic predictions. Earlier in 2018, Nazha et al. highlighted the fact that several prognostic models had been developed for MDS, with the International Prognostic Scoring System (IPSS) and its revised version being the most widely used in clinical practice, and trial eligibility relying mainly on clinical variables from bone marrow and peripheral blood. Incorporating molecular data may enhance predictive accuracy through optimal integration strategies aiming to create personalized prediction models, which could redefine risk stratification in MDS [24]. In the same chronic period, Patel et al. analyzed the use of prognostic models for patients whose disease no longer responds to HMAs (hypomethylating agents). The authors concluded that while conventional prognostic models are useful at baseline, they lose accuracy in the post-HMA failure scenario; specialized models like the post-HMA tool may be more appropriate in that setting. They emphasized the need for further validation, refinement, and possibly the incorporation of molecular markers to enhance predictive accuracy [25]. A more recent study presented that the AI-based MDS prediction score (using flow cytometry data) significantly outperformed the traditional Ogata score. The authors actually suggested that this method could become a robust adjunct diagnostic tool, particularly when conventional parameters are inconclusive. One can observe in Figure 1 that patients with pre-MDS stages were equally distributed in group A and group B. Strikingly, an AI-assisted model showed a progressive evolution of the MDS prediction score from a pre-MDS condition (CHIP) to high-risk MDS, suggesting a linear evolution between these different stages [22]. Despite promising results, several barriers limit the clinical implementation of AI in MDS. These include the lack of large, well-annotated datasets, variability in laboratory techniques (e.g., flow cytometry platforms and staining protocols), and limited external validation across independent cohorts. Additionally, the “black-box” nature of many high-performing models raises concerns regarding interpretability and clinical trust. Regulatory challenges and the need for integration into existing clinical workflows further complicate adoption. Addressing these limitations will be essential for translating AI-based tools into routine MDS practice.

Zhu et al. used machine learning (random forests and CART) to enhance the diagnostic utility of the previously published MDS-CBC score by incorporating platelet data specifically, the immature platelet fraction (IPF). They evaluated over 500 cytopenic patients (168 with MDS and 357 controls) and found that two parameters, Ne-WX (a neutrophil dispersion measure) and the IPF, were the strongest discriminators (explaining ~37% and ~33% of diagnostic decisions, respectively). By combining the original MDS-CBC score with an IPF cutoff of 3% and adjusting the score threshold to 0.23, they derived an extended “e-MDS-CBC score” which achieved a sensitivity of ~88.7% and specificity of ~95.8% for MDS diagnosis. They proposed a two-step decision tree (based on MDS-CBC score thresholds and the IPF) to guide whether a smear review is needed, thereby improving diagnostic efficiency and reducing unnecessary reviews [26]. Elshoeibi et al. (2023) [27] provided a comprehensive review of the current applications and future prospects of artificial intelligence (AI) and machine learning (ML) in the diagnosis of myelodysplastic syndromes (MDSs). The authors discussed various AI and ML techniques employed in analyzing peripheral blood smears, bone marrow samples, and flow cytometry data, highlighting their potential to enhance diagnostic accuracy and efficiency. They also addressed the advantages and limitations of these technologies, emphasizing the need for further research and validation to integrate AI and ML tools effectively into clinical practice for MDS diagnosis [27]. Acevedo et al. (2021) [28] introduced DysplasiaNet, a deep learning model designed to automatically identify hypogranulated neutrophils in peripheral blood smears from patients with MDS. The model was trained on a dataset of annotated images and demonstrated high accuracy, sensitivity, and specificity in detecting dysplastic neutrophils, outperforming traditional image analysis methods. This advancement holds promise for enhancing diagnostic efficiency and consistency in MDS assessment [28]. Table 1 summarizes the distinct patterns that emerge from these studies. Image-based data are most effectively analyzed using deep learning approaches such as convolutional neural networks, which excel in detecting morphological abnormalities. In contrast, structured clinical and laboratory datasets are better suited to machine learning models such as random forests, CART (), and ElasticNet, which provide robust performance and, in some cases, improve interpretability. Across studies, integrating multiple data types—including hematological, molecular, and flow cytometry parameters—consistently improves diagnostic and prognostic accuracy. However, limitations such as small cohort sizes, lack of standardization, and limited external validation remain significant barriers to clinical implementation.

## 5. Part B: AI Tools and Potential Use of Oxidative Stress Biomarkers in MDS

The integration of artificial intelligence with analyses of oxidative stress biomarkers in myelodysplastic syndromes (MDSs) presents a novel approach to prognostic assessment and therapeutic stratification. This synergy leverages the power of machine learning algorithms to discern subtle patterns within complex biomarker data, thereby enhancing the precision of diagnosis and informing personalized treatment strategies. Specifically, AI can analyze markers such as malondialdehyde levels, which are significantly elevated in myelodysplastic syndrome patients compared to healthy controls, and lactate dehydrogenase activity, which also shows a distinct increase in MDS, to identify disease progression and treatment response [29]. Furthermore, AI models can incorporate other oxidative stress indicators, such as serum ferritin levels, which correlate with lipid peroxidation in MDS, to provide a more holistic understanding of the disease’s metabolic derangements [29]. The application of AI in this context facilitates the identification of high-risk patients who may benefit from antioxidant therapies or iron chelation, given that iron overload exacerbates oxidative stress in MDS. This approach allows for the development of predictive models that can forecast disease trajectories and therapeutic efficacy based on intricate relationships between various oxidative stress parameters and clinical outcomes. AI’s capacity to process diverse datasets, including flow cytometry parameters, further refines diagnostic accuracy by integrating cellular morphology and immunological markers with biochemical data [27]. Traditional assessment of OS in MDS relies on individual biomarkers, such as reactive oxygen species levels, antioxidant enzyme activity, and markers of lipid or DNA oxidation. However, these markers alone often fail to capture the complex, multifactorial nature of OS in MDS patients [5,30,31,32]. In detail, AI methods (such as neural networks, decision trees, and support vector machines) can be leveraged to classify, predict, and integrate complex redox-related biochemical data, including reactive oxygen/nitrogen species and antioxidant biomarkers, to uncover latent patterns that may not be evident by classical statistical analysis [33].

**Figure 2:**
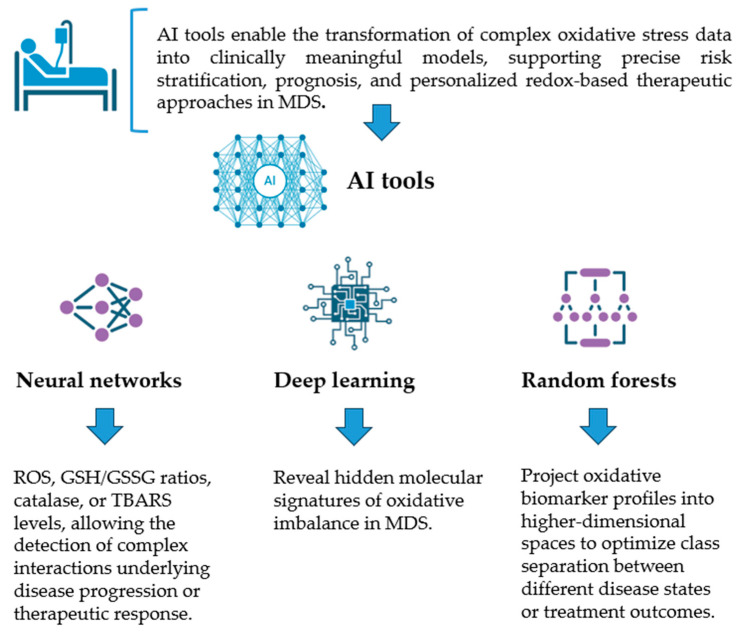
Figure illustration of the AI tools that can be used to transform complex oxidative stress data into clinically meaningful models. Created by the authors using Office 365, PowerPoint.

Neural networks are particularly suited for modeling non-linear and high-dimensional relationships among biomarkers like ROS, GSH/GSSG ratios, catalase, or TBARS levels, allowing for the detection of complex interactions underlying disease progression or therapeutic response. Deep learning variants can integrate multimodal datasets including biochemical, genetic, and transcriptomic data to reveal hidden molecular signatures of oxidative imbalance in MDS. Decision trees, on the other hand, offer high interpretability, generating rule-based models that identify critical thresholds in biomarker values distinguishing stable from progressive MDS. Ensemble methods such as random forests further enhance predictive accuracy and robustness. Support vector machines (SVMs) are effective in handling small or noisy datasets by projecting oxidative biomarker profiles into higher-dimensional spaces to optimize class separation between different disease states or treatment outcomes (Figure 2). However, these markers are typically evaluated individually and do not fully capture the complex, multifactorial nature of oxidative stress in MDS. In this setting, AI-based approaches may offer significant advantages by enabling the integration of multiple oxidative stress parameters with clinical, cytogenetic, and molecular data. Machine learning models including neural networks, decision trees, and support vector machines have shown the capacity in other biomedical contexts to identify non-linear relationships and latent patterns within complex biological datasets. By extension, similar approaches could be applied in MDS to improve biomarker-based risk stratification, disease monitoring, and therapeutic decision-making. Neural networks may be particularly suited for modeling high-dimensional oxidative stress data, whereas decision tree-based methods could provide more interpretable frameworks for clinical use. However, despite this theoretical potential, direct applications of AI specifically targeting oxidative stress biomarker integration in MDS remain scarce. Therefore, future studies are needed to validate these approaches in disease-specific cohorts, develop standardized biomarker panels, and assess their clinical utility. Establishing such evidence will be essential to translate this conceptual framework into practical tools for MDS management.

Collectively, these AI tools enable the transformation of complex oxidative stress data into clinically meaningful models, supporting precise risk stratification, prognosis, and personalized redox-based therapeutic approaches in MDS. The scope should be the training of predictive models on cohorts of MDS patients with known outcomes and identifying the combination(s) of oxidative stress features that could be used to discriminate the disease progression. AI could help turn high-dimensional, noisy redox biomarker data into clinically actionable classifiers, refining prognostic models and paving the way toward personalized redox-informed management in MDS [27]. Taking the data together, AI and ML approaches provide a robust framework for integrating diverse biomarkers with clinical, cytogenetic, and molecular data to enhance diagnostic, prognostic, and predictive analyses [34]. For example, ML algorithms, including random forests, LASSO regression, and Boruta feature selection, can be used to identify the most informative oxidative stress and inflammation markers that correlate with MDS disease severity or progression. Also, the NLR (neutrophil-to-lymphocyte ratio change), oxidative-stress-related metabolites, and inflammatory markers can be combined into composite indices using AI-driven methods for MDS progression. In a recent study, Desideri et al. applied artificial intelligence (AI) in analyzing proteomic data to identify oxidative stress biomarkers in MDS. The study demonstrated how AI models can integrate complex proteomic datasets to uncover specific biomarkers associated with oxidative stress, providing insights into the pathophysiology of MDS and potential therapeutic targets. By linking biological processes with specific oxidative stress biomarkers, the research highlights the potential of AI-driven proteomic analyses in advancing personalized medicine for MDS patients [35]. Although few studies to date have applied AI specifically to analyze oxidative stress biomarkers in MDS, the wealth of data reviewed in Parts A and B, ranging from traditional oxidative stress assays to clinical, molecular, and inflammatory markers, offers a promising foundation for AI-driven analyses. Leveraging these datasets, AI approaches could capture complex, non-linear relationships between oxidative stress biomarkers and clinical outcomes, enabling dynamic risk stratification and potentially allowing earlier intervention for high-risk patients. Furthermore, AI can support the development of interpretable, data-driven decision tools for clinicians. Future directions should focus on large-scale, multicenter studies integrating oxidative stress, molecular, and clinical data, the development of explainable AI frameworks to translate these insights into actionable clinical guidance, and dynamic monitoring of oxidative stress markers to track disease evolution, treatment response, and relapse risk in MDS patients.

## 6. Progress, Obstacles, and Limitations

Recent advances in artificial intelligence have improved biomarker discovery and prognostic modeling through the integration of multi-omics and clinical data, enabling more accurate diagnosis and patient-specific risk stratification in MDS. However, despite this progress, several limitations remain. Many studies are retrospective and lack validation in large, diverse populations, which raises concerns about their generalizability. In addition, most current approaches are still static and do not fully capture the dynamic nature of disease progression. At the same time, important practical and ethical challenges persist, including issues related to data quality and standardization, as well as data privacy and model interpretability. These factors continue to limit the reliable integration of AI into clinical workflows. Overall, while AI offers promising opportunities in this field, further work is needed to address these challenges before its routine clinical application can be fully realized.

## 7. Conclusions

Oxidative stress plays a pivotal role in the pathogenesis and progression of myelodysplastic syndromes, with circulating cells offering a minimally invasive source of clinically relevant biomarkers. However, the complexity and variability of oxidative stress signatures have limited their integration into routine diagnostics. Recent advances in artificial intelligence provide powerful tools to analyze multidimensional biomarker data, identify hidden patterns, and enhance diagnostic precision. AI-driven approaches hold promise not only for improving the early detection and risk stratification of MDS but also for guiding personalized treatment strategies and monitoring disease progression. Among current approaches, random forest and Gradient Boosting models appear to be the most consistently robust for structured clinical and biomarker data, while convolutional neural networks remain the preferred choice for image-based analysis. To translate this potential into clinical practice, future efforts must focus on generating large, high-quality datasets, standardizing biomarker assessment protocols, and addressing ethical and regulatory challenges. Ultimately, the synergy between oxidative stress biomarker research and AI technologies could transform diagnostic workflows in MDS, enabling earlier intervention and better patient outcomes.

## Figures and Tables

**Table 1:** Summary of the most important findings of PART A.

Type of Article	Data Type	AI-Techniques and Other	Result	Next Step	Limitations	Clinical Relevance
Original [26]	Hematological parameters (CBC, IPF)	Machine learning (random forests and CART)	Found that two parameters, Ne-WX (a neutrophil dispersion measure) and IPF, were the strongest discriminators	Propose a two-step decision tree (based on MDS-CBC score thresholds and IPF) to guide whether a smear review is needed	Limited to specific parameters; requires validation	May reduce unnecessary smear reviews and improve diagnostic efficiency
Original [24]	Clinical + molecular data	Prognostic models	Incorporating molecular data may enhance predictive accuracy through optimal integration strategies	To create personalized prediction models	Lack of standardized integration strategies	Supports the development of personalized prognostic tools
Original [22]	Flow cytometry	AI-based MDS prediction score (using flow cytometry data)	Outperforms the traditional Ogata score. AI empowers the diagnosis of myelodysplastic syndromes by multiparametric flow cytometry	Suggest that this method could become a robust adjunct diagnostic tool	Requires specialized datasets and validation	Promising adjunct diagnostic tool, especially in unclear cases
Review [27]	Mixed (imaging, clinical, flow cytometry)	Artificial intelligence, machine learning, and deep learning	A significant proportion of the studies examined exhibited excellent predictive capabilities, with an AUC greater than 0.9.	Utilization of machine learning algorithms holds significant promise in the diagnosis of MDS	Heterogeneity across studies; lack of standardization	Confirms potential of AI in MDS diagnosis
Original [28]	Peripheral blood smear images	Convolutional neural networks	Primary contribution of this work is a predictive model for the automatic recognition in an objective way of hypogranulated neutrophils in peripheral blood smear	The utility of the model implemented is as an evaluation tool for MDS diagnosis integrated in the clinical laboratory workflow	Requires large, annotated datasets; less interpretable	Enhances diagnostic consistency and automation

## Data Availability

No new data were created or analyzed in this study.
